# Study on the Size Dependence of the Shell-Breaking Response of Micro/Nano Al Particles at High Temperature

**DOI:** 10.3390/nano14030265

**Published:** 2024-01-26

**Authors:** Zhengqing Zhou, Lujia Chai, Tianyi Wang, Huiling Jiang, Zhiming Bai, Wenbo Yuan, Jinguo Sang

**Affiliations:** 1 Research Institute of Macro-Safety Science, University of Science and Technology, Beijing 100083, China; zhouzhengqing@ustb.edu.cn (Z.Z.); chailj13598022610@163.com (L.C.); 15510298559@163.com (T.W.); 2 School of Civil and Resource Engineering, University of Science and Technology Beijing, Beijing 100083, China; 3 Shandong Jinruan Science and Technology Co., Ltd., Yantai 265400, China; ywb@zhaojin.com.cn (W.Y.); sang_21@139.com (J.S.)

**Keywords:** Al particle, shell-core structure, thermal stress, shell breaking

## Abstract

The reactivity of Al nanoparticles is significantly higher than that of micron Al particles, and the thermal reaction properties exhibit notable distinctions. Following the previous studies on micron Al particles, the shell-breaking response of Al nanoparticles under vacuum conditions was analyzed using COMSOL simulation. Relationships between thermal stabilization time, shell-breaking cause, shell-breaking response time, and particle size were obtained, and a systematic analysis of the differences between micrometer and nanometer-sized particles was conducted. The results indicate that the thermal stabilization time of both micrometer and nanometer particles increases with the enlargement of particle size. The stress generated by heating Al nanoparticles with sizes ranging from 25–100 nm is insufficient to rupture the outer shell. For particles within the size range of 200 nm to 70 μm, the primary cause of shell-breaking is compressive stress overload, while particles in the range of 80–100 μm experience shell rupture primarily due to tensile stress overload. These results provide an important basis for understanding the shell-breaking mechanism of microns and nanoparticles of Al and studying the oxidation mechanism.

## 1. Introduction

Aluminum (Al) powder finds widespread application in explosives and propellants owing to its exceptional reactivity and high energy density [1,2,3,4,5]. Al nanoparticles in their natural state have an amorphous alumina shell [6,7], and the activity of alumina is much lower than Al, so the oxide shell protects the internal Al from further oxidation but also hinders the energy release of the particles. In addition, Al powder is highly flammable and inevitably generates dust during industrial processes [8,9,10,11]. Upon reaching its ignition temperature, the Al powder undergoes combustion, rupturing the oxide shell and exposing the internal Al core to ambient air, culminating in an explosive event. Therefore, it is of great significance to study the rupture mechanism of the oxidized shell, both in terms of improving the energy release rate and ensuring industrial safety.

Numerous studies have been conducted to investigate the causes of the rupture of Alumina shells. Zhou et al. [12] characterized the physicochemical properties of micron Al particles before and after the reaction in the air using a scanning electron microscope and a laser particle size analyzer. The experiments found that the rupture of the oxide shell occurred after the melting of the internal Al, and the expansion of the molten Al liquid was one of the factors affecting the rupture of the shell. Hou et al. [13] used a high-speed microscope to record the combustion process of Al nanoparticles under laser ignition, and the results also showed that the thermal expansion of Al nanoparticles leads to the rupture of the alumina shell, and the molten Al core overflows and evaporates, resulting in ignition and combustion. Sun et al. [14] used thermogravimetric analyzers and differential scanning calorimetry to study the thermal reaction properties of nanoscale and microscale Al nanoparticles in a CO_2_ environment. Their findings revealed that nanoscale Al particles were mainly oxidized by CO_2_ diffusion, that oxidation occurred, and the oxide shell kept thickening but did not rupture, while the pressure gradient due to the expansion of the micron Al powder caused the cracking of the oxide shell. The melt-dispersion mechanism (MDM) proposed by Levitas et al. [15,16] also suggests that the increased pressure in the nucleus due to the volume expansion of the melted Al nucleus was the reason for the rupture of the oxide layer. However, it only applied to the case at high heating rates and not at low heating rates.

Some researchers have argued that oxide shell rupture is also affected by alumina crystalline transformation. Trunov’s study [17,18] showed that alumina undergoes amorphous, γ, and α (θ) alumina transformations sequentially at high temperatures. The densities of these alumina oxides progressively increase, and thus the alumina shells become denser and less voluminous when heated and the shells shrink leading to rupture. Rai [19] analyzed the oxidation of Al nanopowder using transmission electron microscopy and mass spectrometry and the results similarly support the notion that alumina crystal transformation leads to the rupture of the oxide shells. Sundaram [20] established a general theory for the ignition and combustion of Al particles at the nano- and micrometer scale, and concluded that the cracking of the alumina shells is a result of a combination of internal stresses and crystal transformation. Savel’ev [21] developed a model to investigate the role of polycrystalline transformation of Al nanoparticles in the ignition process. The results showed that the polycrystalline transformation of amorphous alumina to γ-alumina always precedes the ignition of nanoparticles, and the ignition of particles occurs after the cracks of γ-alumina films heal during the growth phase, while the polycrystalline transformation of γ-oxides to α-oxides does not have a significant effect on the ignition of nanoparticles.

In summary, there are many studies on the thermal oxidation behavior of Al particles in oxygen, carbon dioxide, and other atmospheres, but the shell rupture mechanism of alumina shells is still not uniformly explained. Although shell breaking is affected by intra-particle stresses, the stresses are seldom quantitatively characterized, and the thermal stabilization time of the particles and the shell-breaking time are rarely mentioned. The small size effect and surface effect of Al nanoparticles have led to higher reactivity than micron Al particles [22,23], and thus to a wider range of applications, but have also led to a different reaction mechanism than that of micron Al particles. The previous paper [24] investigated the force-heat coupling process of micron particles and analyzed the shell-breaking characteristics and causes, and this paper takes up the previous paper to quantitatively characterize the thermo-mechanical behavior of Al nanopowder using simulation software COMSOL. Oxide shell rupture is affected by a variety of zfactors [25] such as internal stress due to differences in coefficients of thermal expansion, oxidative thickening of the shell, and crystal transformation, etc. To exclude the effect of oxidation on shell-breaking, the thermal behavior of nanoparticles during the vacuum process was investigated. The reaction characteristics of micron and nano Al powder were compared from three aspects: thermal stabilization time, stress distribution and magnitude, and shell-breaking characteristics. And the shell-breaking mechanism of micron and nano Al powder was systematically analyzed.

## 2. Finite Element Model

**Figure 1:**
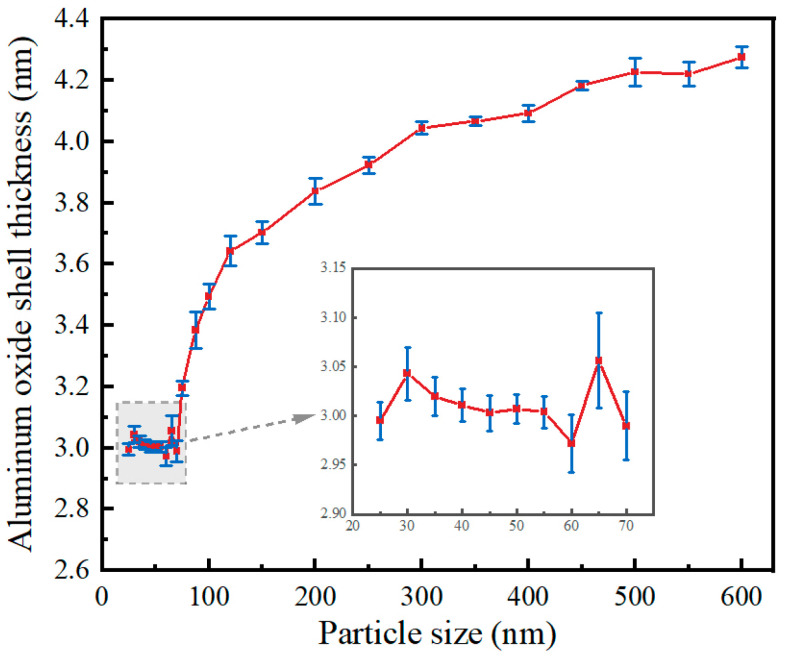
Relationship between oxide shell thickness and particle size of 25–600 nm Al nanoparticles [26].

**Figure 2:**
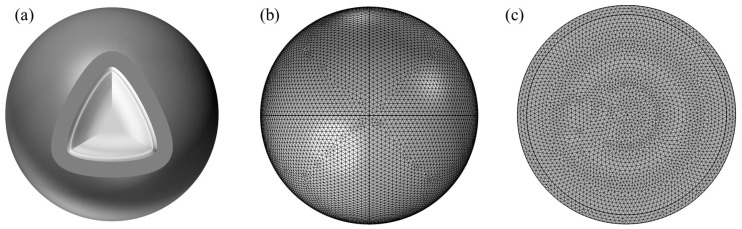
Model of Al nanoparticles, (**a**) Shell-core model diagram, (**b**) Mesh division of oxide shell, (**c**) Mesh division of Al core.

Simulation software COMSOL (version 6.2) based on the finite element method, through the solution of partial differential equations (individual field) or partial differential equations (multi-field) to realize the physical phenomena, can be realized arbitrarily using multi-physics field direct coupling analysis. To further study the cracking kinetics of the oxide shell of an individual Al nanoparticle in a 650 °C vacuum environment, this section employs the multi-physics simulation software COMSOL for accurate simulation. The research by Zeng et al. [26] has shown that the relationship between the oxide shell thickness and particle size of Al nanoparticles is different in the size ranges of 25 to 70 nm and 100 to 600 nm, as shown in Figure 1. Therefore, shell-breaking models were established for Al nanoparticles in the size ranges of 25–70 nm and 100–600 nm, respectively. The core-shell model of Al nanoparticles and the corresponding mesh division are shown in Figure 2a, b and c, respectively.

The solid heat transfer and thermal expansion equations built into COMSOL were used as control equations for Al and alumina, and the solid heat transfer control equation and thermal expansion equation are shown in Equations (1) and (2), respectively.

(1) $$\rho C_{P}\frac{\partial T}{\partial t}=Q+\nabla \cdot (k\nabla T)$$

where ρ is the density, $C_{P}$ is the constant pressure heat capacity, T is the temperature, t is time, Q is the absorbed heat, ∇ is the Laplace operator, and k is the coefficient of thermal conductivity.

(2) $$E_{\mathrm{th}}=\alpha (T-T_{0})$$

where $E_{\mathrm{th}}$ is the thermal strain, α is the coefficient of thermal expansion, $T_{0}$ is the initial temperature, and T is the current temperature.

Al nanoparticles mainly contain Al in the core and alumina in the outer layer, and the main physical properties of Al and alumina are shown in Table 1.

**Figure 3:**
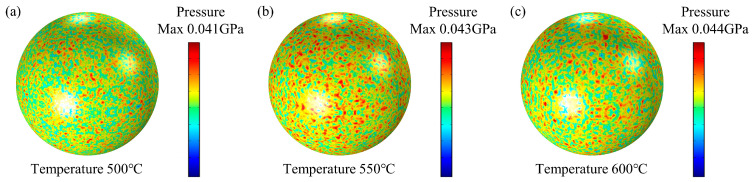
The cloud view of the maximum pressure on Al nanoparticles at different temperatures calculated by the present model. (**a**) 500 °C, (**b**) 550 °C, (**c**) 600 °C.

Firmansyah et al. [29]. investigated the relationship between the Al lattice and temperature of Al nanoparticles with a diameter of ~100 nm, and calculated the pressure generated within the Al core. The results showed that the maximum pressure in the Al core of 100 nm Al nanoparticles at 600 °C was 0.051 Gpa (See Ref. [30] Figure 5b). To verify the accuracy of the Al particle model with this result, an Al nanoparticle model with a diameter of 100 nm and an alumina shell thickness of 4.6 nm was established. The simulation results indicate that the maximum compressive stress of the Al core at 600 °C is 0.044 GPa, as shown in Figure 3. The error in the simulation results was calculated to be 13.72%.

## 3. Results and Discussion

### 3.1. Heating Process of Al Nanoparticles

**Figure 4:**
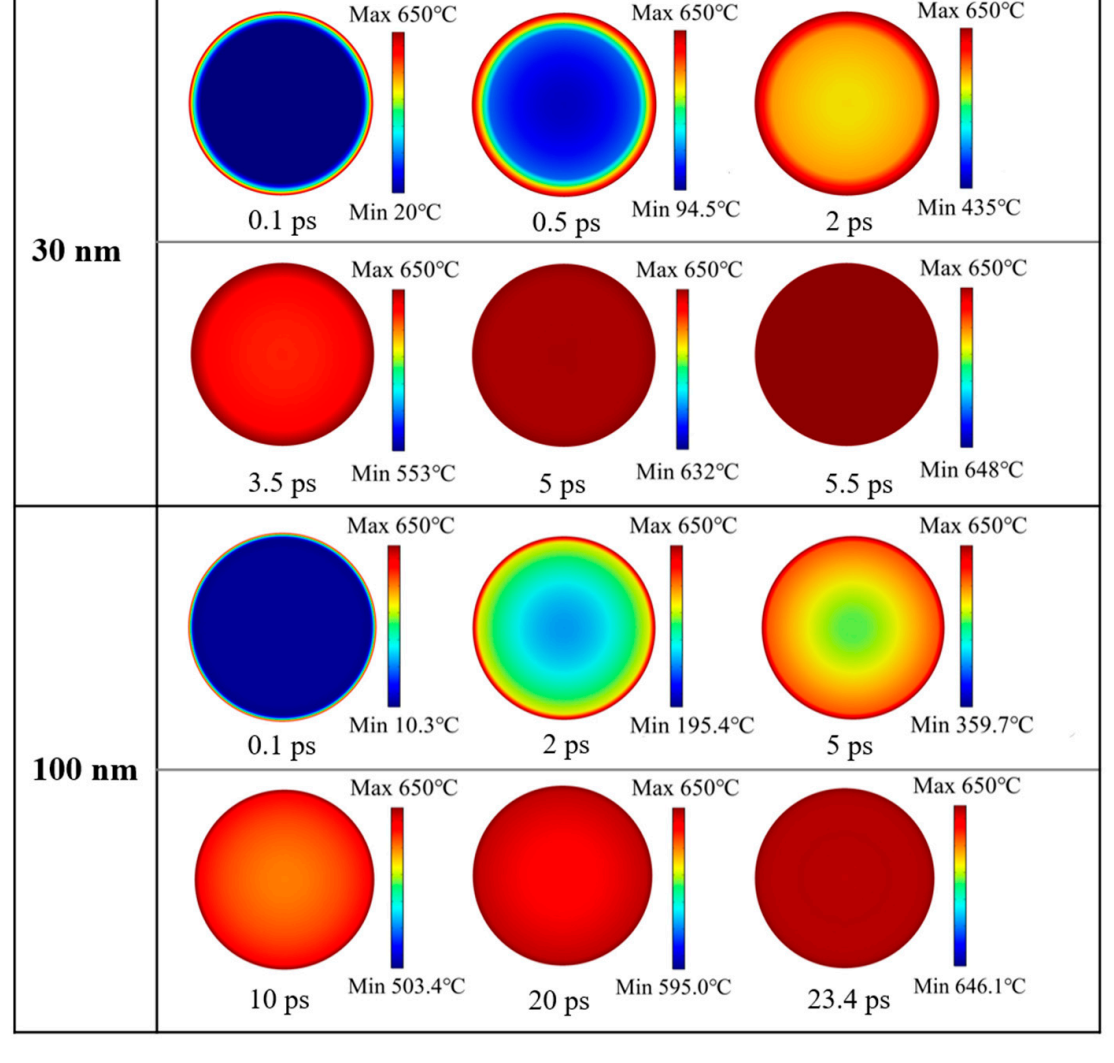
Temperature distribution of 30 and 100 nm Al nanoparticles at different times.

**Figure 5:**
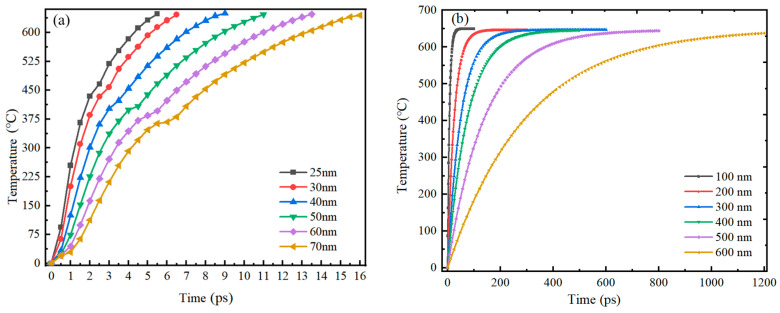
Center temperature versus time for Al nanoparticles of different sizes. (**a**) 25–70 nm. (**b**) 100–600 nm.

Figure 1 reveals that there is a great difference in shell thickness versus particle size for Al nanoparticles of 25–70 nm and 100–600 nm. And the simulation results showed that the heating of 25–70 nm particles is basically the same, and that of 100–600 nm particles is basically the same. Therefore, 30 nm and 100 nm particles were selected as representatives from these two categories for demonstration, respectively. The ambient temperature was 650 °C and the initial temperature of the Al nanoparticles was 0 °C. The overall temperature of the Al nanoparticles increased gradually with time. The distribution of temperatures at the center of the 30 nm and 100 nm Al nanoparticles as a function of time is shown in Figure 4. When the particles are heated, the alumina shell is first heated to a higher temperature, and then heat is gradually transferred to the Al core until the temperature inside and outside the particles is consistent. Figure 5 demonstrates the center temperature of different sized particles as a function of heating time. From Figure 4 and Figure 5, it can be seen that the increase in temperature of Al nanoparticles is not a uniform process. For Al nanoparticles of 25–70 nm, the center temperature inside the particles increases rapidly and then slowly; for Al nanoparticles of 100–600 nm, the center temperature inside the particles increases rapidly and then tends to stabilize. And the larger the particle size, the slower the rate of temperature increase.

**Figure 6:**
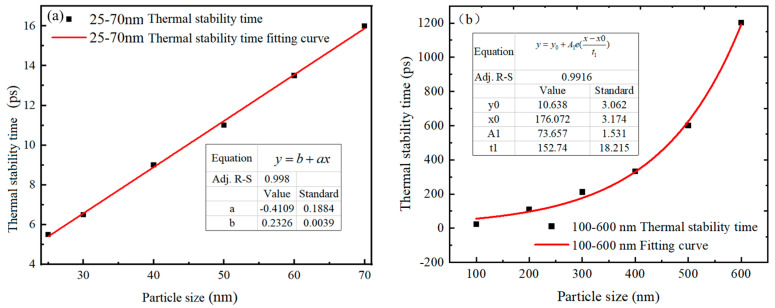
Thermal stability time versus particle size for Al nanoparticles of different sizes. (**a**) 25–70 nm. (**b**) 100–600 nm. (The thermal stability time refers to the time required for the temperature at the center of the particle to reach the ambient temperature).

The thermal stabilization time of 25–70 nm and 100–600 nm Al nanoparticles is shown in Figure 6. It is observed that the thermal stabilization time of Al nanoparticles increases with the increase of particle size, regardless of the particle size. From Figure 6a, it can be seen that the thermal stabilization time of Al nanoparticles of 25–70 nm is linearly related to the particle size, and the thermal stabilization time of these particle sizes is between 5.404–15.971 ps. The fitting function between the thermal stabilization time and the particle size is shown in Equation (3).

(3) t=0.2326d−0.4109,25 nm≤d≤70 nm

where *t* is the thermal stability time (ps), and *d* is the particle size of the Al nanoparticles (nm). It is noteworthy that although the thermal stabilization times of Al nanoparticles with different particle sizes are different, the trends of the center temperatures are basically the same. This indicates that for 25–70 nm Al nanoparticles, the particle size does not affect the heat conduction and temperature diffusion.

However, for 100–600 nm Al nanoparticles, the relationship between thermal stabilization time and particle size showed no linear increase, but rather a trend of slow increase followed by rapid increase (shown in Figure 6b). It can be seen that the larger the particles, the slower the heat conduction speed, and the longer it takes for the Al nanoparticles to be completely heated. The thermal stability time for Al nanoparticles with diameters above 100 nm is 23.4–1203.1 ps, and the fitted function of the thermal stability time with respect to the particle size is expressed as Equation (4).

(4) $$t=73.657e(\frac{d-176.072}{152.74})+10.638,100\;\mathrm{nm}\leq d\leq 600\;\mathrm{nm}$$

where *t* is the thermal stability time (ps), and *d* is the particle diameter of Al (nm).

### 3.2. Thermal Stress of Al Nanoparticles

#### 3.2.1. Compressive Stress Distribution on Alumina Shells

**Figure 7:**
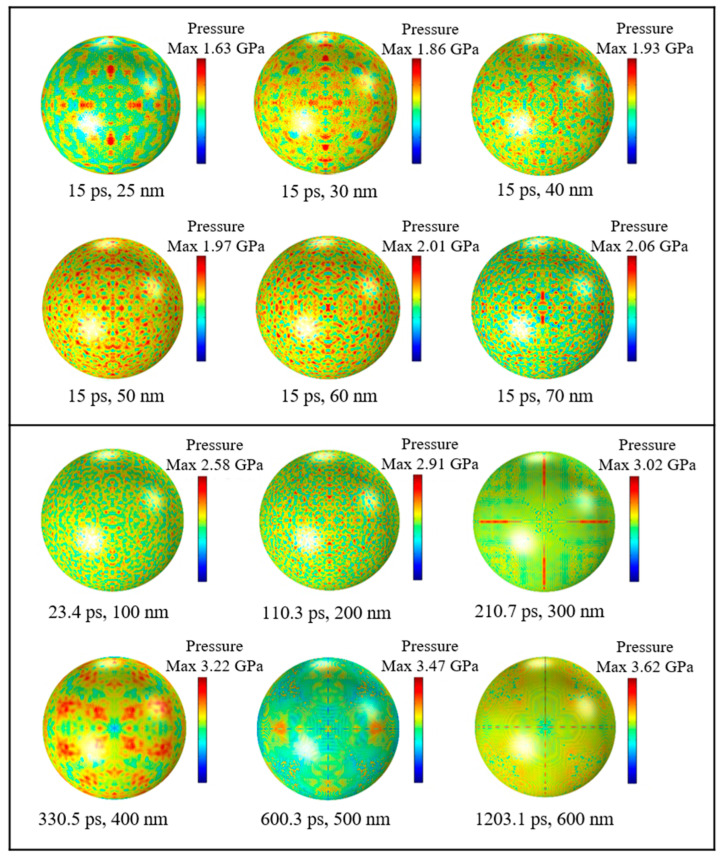
Compressive stress distribution of Al nanoparticles of different sizes at thermal stability time.

To figure out the distributions and numerical magnitudes of compressive stresses in Al nanoparticles with different particle sizes, simulation tests were carried out on Al nanoparticles of different particle sizes, respectively. Figure 7 shows a cloud view of the compressive stress distribution on the outer surface of the alumina shell for Al nanoparticles of different sizes. It can be seen that when the diameter of the particles is less than 100 nm, the maximum compressive stress is basically uniformly distributed on the shell in the form of a dot. This uniform distribution indicates that the alumina shell of Al nanoparticles has good compressive strength and can withstand a certain pressure in a high temperature environment. The maximum compressive stress of Al nanoparticles in the range of 100 to 200 nm is also uniformly distributed in a point shape, whereas the Al nanoparticles in the range of 300 to 600 nm are distributed on the surface of the shell in the form of flakes and two perpendicular to each other straight lines, and the pressure is concentrated in localized areas. There is an obvious difference in the compressive stress distributions of Al nanoparticles in the two ranges due to the fact that the Al nanoparticles have significant differences in physical properties such as surface area and the R ratio between Al nanoparticles ranging from 100 to 300 nm and 400 to 600 nm, which result in different thermal stresses and thermal pressures during the thermal stability time.

**Figure 8:**
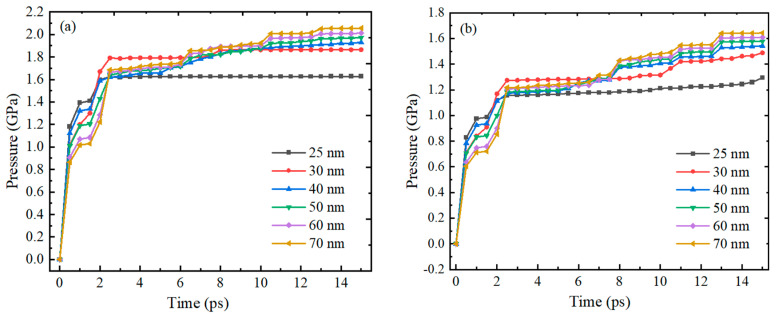
The relationship between the compressive stress on the inner and outer surfaces of the Al oxide shell of Al nanoparticles with particle sizes of 25–70 nm and time. (**a**) Outer surface (**b**) Inner surface.

Figure 8 shows the trend of compressive stress on the inner and outer surfaces of the Alumina shell of Al nanoparticles with particle sizes of 25–70 nm over time. The results show that the trend of compressive stress change on the inner and outer surfaces of the Alumina shell of Al nanoparticles is basically consistent over time, both increasing rapidly within 2 ps and then gradually stabilizing, with slight fluctuations. The basic consistency of the trend in the change of compressive stress on the inner and outer surfaces of the alumina shell also indicates that there is good coupling between the inner and outer surfaces of the Alumina shell of the Al nanoparticles during thermal expansion. Although there are still slight fluctuations during the stabilization process, these fluctuations do not affect the overall stability of the Alumina shell of the Al nanoparticles.

**Figure 9:**
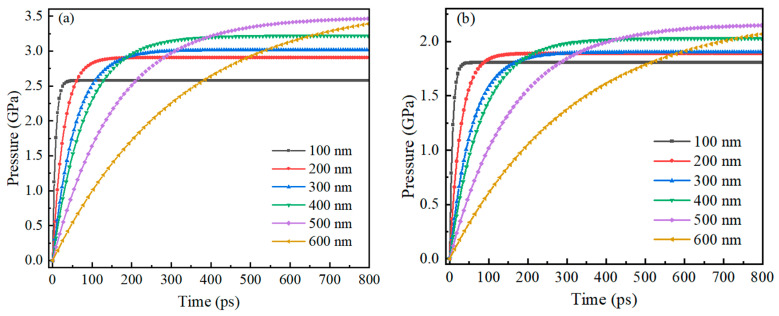
The relationship between the compressive stress on the inner and outer surfaces of the Alumina shell of Al nanoparticles ranging from 100 to 600 nm and time during oxidation. (**a**) Outer surface (**b**) Inner surface.

Figure 9 shows the trend of compressive stress on the inner and outer surfaces of the Alumina shell of Al nanoparticles ranging from 100 to 600 nm with time during oxidation. The research results also show that the trend of pressure on the inner and outer surfaces of the Alumina shell of Al nanoparticles is basically the same, with rapid increase followed by gradual stabilization. In addition, the time required for rapid pressure increase is basically consistent with the thermal stability time of Al nanoparticles, indicating that the change in pressure field is due to the change in temperature field.

#### 3.2.2. Tensile Stress Distribution on Alumina Shells

**Figure 10:**
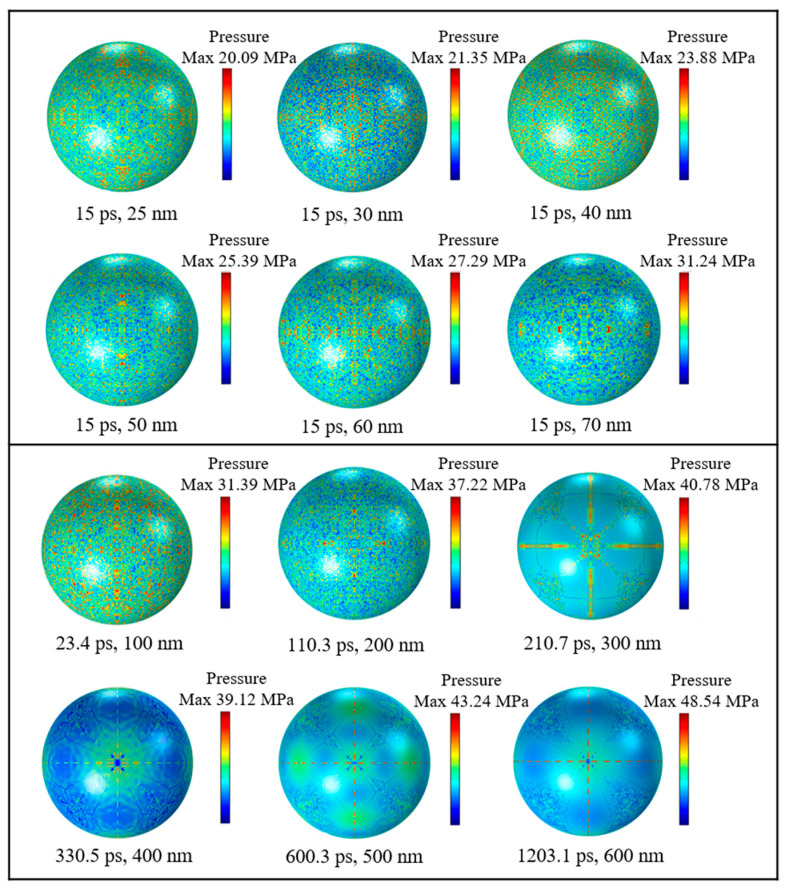
Tensile stress distribution of Al nanoparticles of different sizes during thermal stability time.

Figure 10 shows the cloud diagram of tensile stress distribution on the outer surface of alumina shell for Al nanoparticles of different sizes. The distribution of tensile stress extremes of Al nanoparticles with a particle size of 25–70 nm are basically uniformly distributed on the alumina shell in the shape of dots, and it can be seen that the small particle size has almost no effect on the distribution of tensile stress extremes, indicating that when the diameter is less than 100 nm, the particle size does not have much effect on the mechanical properties of the alumina shell of Al nanoparticles. And the compressive stress demonstrates the same phenomenon, which further indicates that the compressive and tensile stresses in the Al particle alumina shells come from the same source, i.e., the temperature field variation.

In the range of 100–600 nm, due to the difference of specific surface area and R ratio, the maximum tensile stress of 100–200 nm Al nanoparticles is uniformly distributed (which is consistent with the distribution of 25–70 nm), and the maximum tensile stress of 300–600 nm Al nanoparticles is distributed in two straight lines perpendicular to each other. It is worth noting that in the previous article on micrometer Al particles [24], the maximum stress is mainly distributed in two lines at 45° and 135°, which are also two straight lines perpendicular to each other, and this is consistent with the distribution of the stress in 300–600 nm in this paper. Therefore, it can be considered that the maximum stress at 25–70 nm is uniformly distributed, while the maximum stress at 100–200 nm is uniformly distributed but begins to transition towards a vertical linear distribution. The maximum stress at 300–600 nm becomes a vertical linear distribution, and the maximum stress of 10–100 μm is also a vertical linear distribution.

**Figure 11:**
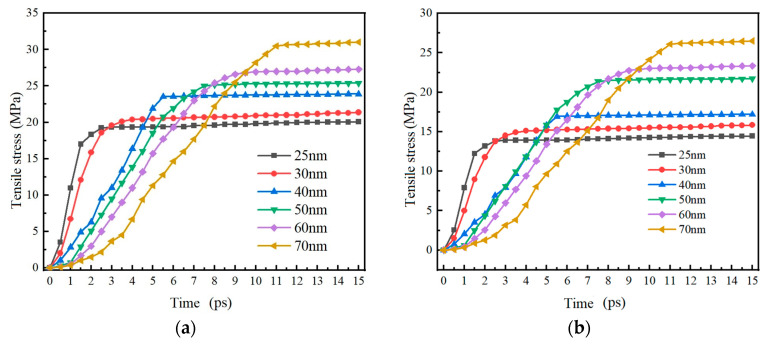
The relationship between tensile stress on the inner and outer surfaces of the oxide shell of Al nanoparticles with particle sizes of 25–70 nm and time. (**a**) Outer surface (**b**) Inner surface.

Figure 11 shows the trend of tensile stress on the inner and outer surfaces of the Alumina shell of Al nanoparticles with particle sizes of 25–70 nm over time. The research results show that the trend of tensile stress change on the inner and outer surfaces of the Alumina shell of Al nanoparticles is basically consistent over time, both increasing rapidly within 11 ps and then gradually stabilizing. Similar to the compressive stress trend study above, the tensile stress on the Alumina shell of Al nanoparticles also shows good stability. It is worth noting that the time required for rapid increase in tensile stress is basically the same as the thermal stability time of the Al nanoparticles, and is much longer than the time required for rapid increase in compressive stress. This result indicates that the Alumina shell of Al nanoparticles responds to tensile stress at a slower speed in a high-temperature environment, requiring some time to adapt and stabilize.

**Figure 12:**
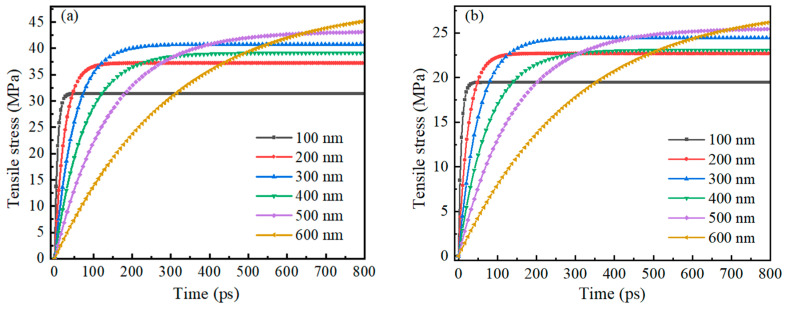
The relationship between the change of tensile stress on the inner and outer surfaces of the Alumina shell of Al nanoparticles ranging from 100 to 600 nm and time during oxidation. (**a**) Outer surface (**b**) Inner surface.

Figure 12 shows the trend of tensile stress on the inner and outer surfaces of the Alumina shell of Al nanoparticles ranging from 100 to 600 nm with time during oxidation. As with the 25–70 nm Al nanoparticles, the tensile stresses on both the inner and outer surfaces of the shells increase rapidly and then stabilize gradually, again showing good stability. The time required for rapid increase in tensile stress is basically consistent with the thermal stability time of Al nanoparticles, which is similar to the time required for rapid increase in pressure. This result indicates that in a high-temperature environment, the response rate of the Alumina shell of Al nanoparticles ranging from 100 to 600 nm to pressure and tensile stress is basically the same.

#### 3.2.3. Comparison of Compressive Stress and Tensile Stress

**Figure 13:**
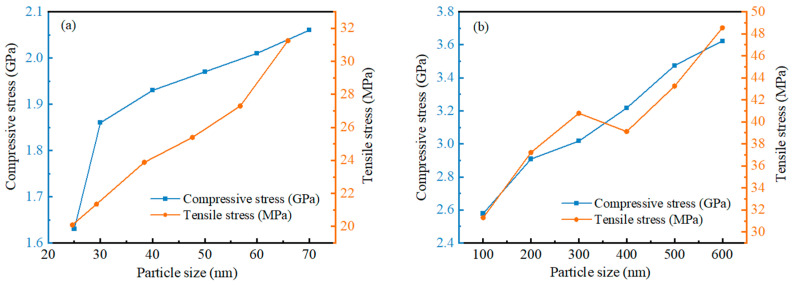
The relationship between the maximum compressive stress and maximum tensile stress on alumina shells with different sizes of Al nanoparticles. (**a**) 25–70 nm. (**b**) 100–600 nm.

The relationship between maximum compressive stress and maximum tensile stress is different for different particle size ranges. Figure 13 shows the relationship between maximum compressive stress and maximum tensile stress on the outer surface of alumina shells for different sizes of Al nanoparticles with respect to particle size. The results show that the maximum compressive stress increases sharply at a particle size of 30 nm in the 20–70 nm particle size range, but increases slowly after 30 nm, showing an essentially linear relationship. In contrast, the maximum tensile stress does not increase sharply, but shows a basically stable linear growth trend, and the value of tensile stress is three orders of magnitude smaller than that of compressive stress. This suggests that the alumina shells of Al nanoparticles with a particle size of 25–70 nm undergo significant deformation and changes when subjected to compressive stresses, while exhibiting a relatively stable response when subjected to tensile stress. Whereas in the 100–600 nm particle size range, the maximum compressive stress shows a relatively stable linear increase, the maximum tensile stress shows a different trend. Specifically, the maximum tensile stress increases rapidly in the range of 100 to 300 nm; after a slow decrease around 400 nm, it continues to increase rapidly. This result indicates that the alumina shells of Al nanoparticles in the range of 100 to 600 nm under tensile stress undergo significant deformation and changes, while they show a relatively stable response under compressive stresses.

### 3.3. Breaking Characteristics and Mechanism of Al Nanoparticles

**Figure 14:**
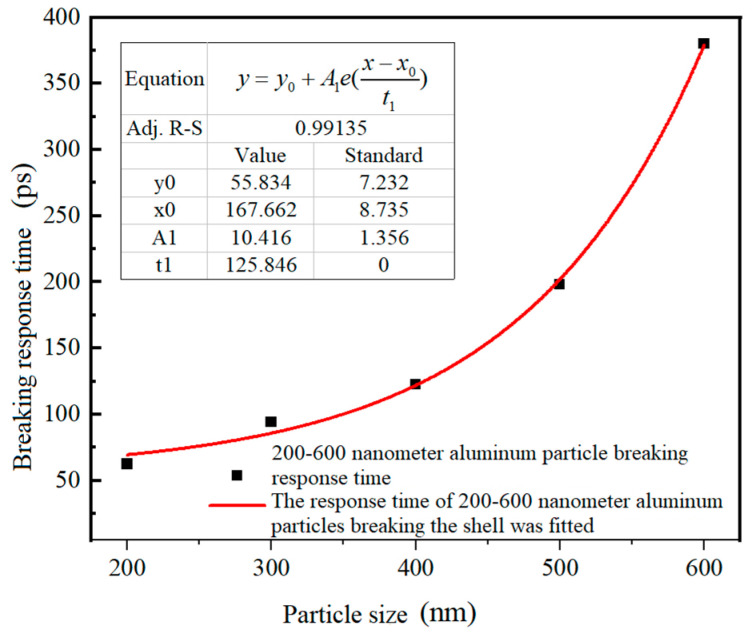
The relationship between shell-breaking response time and particle size of Al nanoparticles with particle sizes of 200–600 nm.

Table 2 shows the shell-core relationship, compression shell-breaking response time, tensile shell-breaking response time, and direct cause of shell-breaking response of Al nanoparticles with different sizes. The tensile strength of alumina is between 35.5 and 53.1 MPa [31]. In this paper, 35.5 MPa was used as the tensile strength of alumina, and the compressive strength of alumina is 2600 MPa [30]. The time required for compressive stress to reach compressive strength is the compressive stress shell-breaking response time. Similarly, the time required for tensile stress to reach the tensile stress strength is the tensile shell-breaking response time. The smaller value of the two is the shell-breaking response time. Since the Al nanoparticles of 25–100 nm heated at 650 °C could not reach the values of compressive and tensile strength of alumina, no compression damage or tensile damage occurred to their alumina shells. In contrast, for Al nanoparticles of 200–600 nm, the stress induced by the temperature of 650 °C can rupture the alumina, and the compressive stress overload is the main reason for the rupture of the oxide shell. Figure 14 shows the relationship between the shell-breaking response time and particle size for 200–600 nm Al nanoparticles, and it can be seen that the larger the particle size, the longer the shell-breaking response time. However, the relationship between shell-breaking response time and particle size does not increase linearly, but is a gradually accelerating process. The fitting function of the shell-breaking response time to particle size is shown in Equation (5).

(5) $$t=10.416e(\frac{d-167.662}{125.846})+55.834,200\;\mathrm{nm}\leq d\leq 600\;\mathrm{nm}$$

where, *t* is the shell-breaking response time (ps), and *d* is the particle size of Al nanoparticles (nm).

### 3.4. Comparative Analysis of Micro and Nano Al Particles

#### 3.4.1. Temperature Distribution and Thermal Stabilization Time

**Figure 15:**
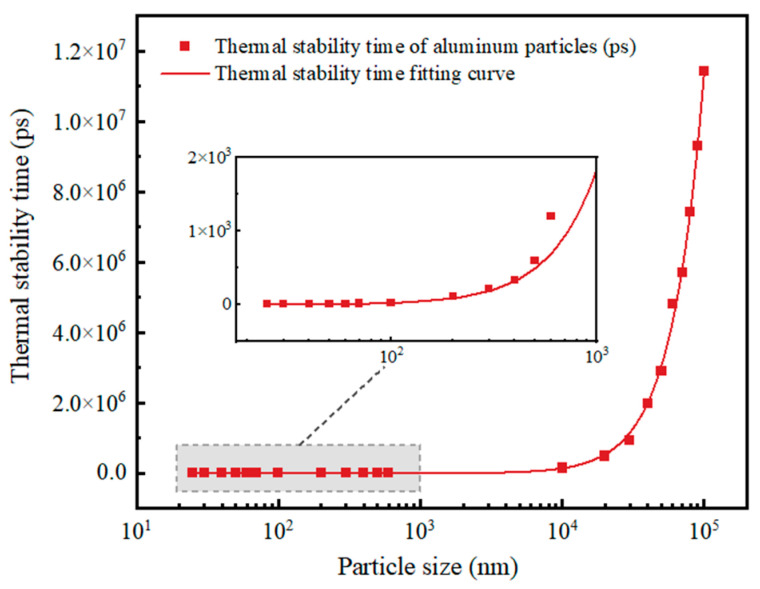
Thermal stabilization time of Al particles as a function of particle size.

According to the previous article and the results of this paper, no matter whether they are micron or Al nanoparticles, the larger the particle size, the slower the particles are heated and the longer the thermal stabilization time; the change trend is shown in Figure 15. The warming trends of individual particles are all rapidly rising and then leveling off. The difference is that the thermal stabilization time of Al nanoparticles of 100 nm and above is nonlinear with particle size, while the thermal stabilization time of Al nanoparticles of 25–70 nm is linear with particle size. This is most likely because the thickness of the oxide shell of the Al nanoparticles of 25–70 nm has no relationship with the particle size, which always stays around 3 nm. Moreover, according to the obtained thermal stabilization time, the time required for the micron Al powder to be completely heated is three orders of magnitude higher than that of the nano Al powder, which also indicates that, in a high temperature environment, the nano Al powder can break the shells and release the energy faster than the micron Al powder, thus improving the energy release rate of the explosives.

#### 3.4.2. Stress Distribution and Variation

**Figure 16:**
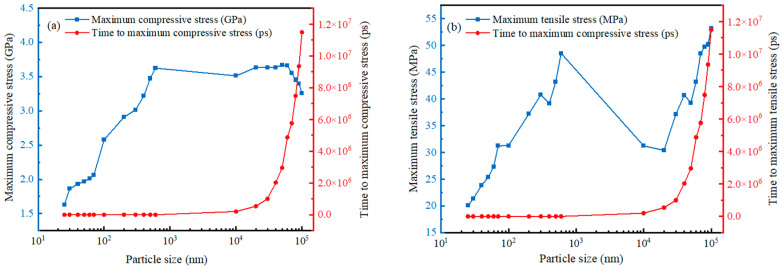
Variation curves of the maximum pressure and the time to reach the maximum pressure for different sizes Al particles. (**a**) Maximum compressive stress. (**b**) Maximum tensile stress.

For the stress situation of the alumina shell, no matter the compressive stress or tensile stress, in general, the maximum stress at 25–70 nm is uniformly distributed, while the maximum stress at 100–200 nm is also uniformly distributed but begins to transition towards a vertical linear distribution. The maximum stress at 300–600 nm becomes a vertical linear distribution, and the maximum stress of 10–100 μm is also a vertical linear distribution. The pressure on the inner surface of the alumina shell and the outer surface of the pressure tends to increase rapidly and then stabilize, but the pressure on the outer surface is always higher than the pressure on the inner surface, which is due to the change of the stress originated from the change of the temperature field. There is a distance between the inner surface and the outer surface corresponding to the shell thickness, and the temperature fields of the inner surface and the outer surface need a period of time to reach equilibrium. With the stabilization of the temperature field, the stress tends to be stabilized, so that the increase of the inner surface pressure will be lagged. In addition, as the particle size increases, the maximum pressure on the alumina shell increases, and the pressure size of the micron Al powder is three orders of magnitude larger than that of the nanometer Al powder. Figure 16 illustrates the maximum pressure applied to the micro and Al nanoparticles and the time to reach the maximum pressure. It can be seen from the figure that the time to reach the maximum pressure on the alumina shells always increased gradually with increasing particle size, regardless of whether it is micron or nanoparticles, compressive or tensile stresses. The change in maximum pressure also basically increased gradually with increasing particle size, but the change in compressive stress for micron particles exhibited the opposite trend. Starting from 10 μm, the maximum compressive stress increased extremely slowly with increasing particle size and started to decrease when the particle size exceeded 60 μm. This is due to the fact that the maximum compressive stress is determined by both the center temperature and the ratio R of the Al particles. The lower the center temperature, the smaller the maximum compressive stress and the larger the ratio R [24].

#### 3.4.3. Characteristics and Mechanism of Shell Breaking

**Figure 17:**
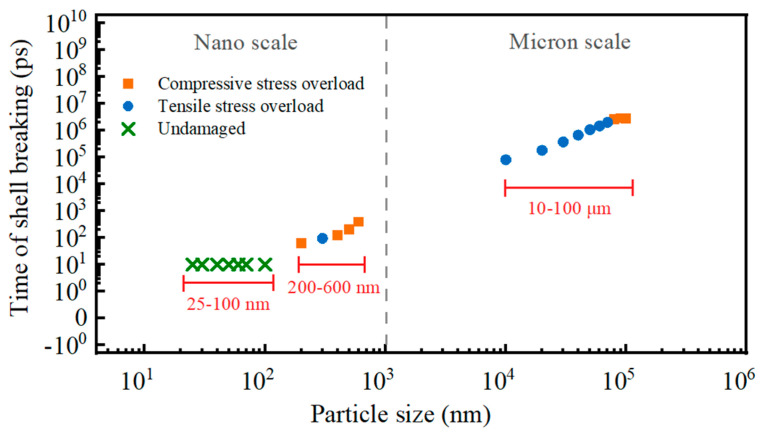
Shell breakage time and reasons of Al particles with different sizes.

The shell-breaking characteristics and mechanisms of Al particles of different sizes are different. When the alumina is pressed to its ultimate pressure, all the micron Al particles break their shells, and the reason for 10–70 micron Al particles to break their shells is the compressive stress overload, and the reason for 80–100 micron Al particles to break their shells is the tensile stress overload. Nanoparticles are different from micron particles in that 25–100 nm Al particles are not damaged because the current temperature (i.e., 650 °C) does not allow the particles to be stressed to the stress extremes of alumina. While 200–600 nm Al particles break their shells, and the main reason is compressive stress overload. The reason for shell breakage and response time of shell breakage of micro and Al nanoparticles are shown in Figure 17. It can be seen that the larger the particle size, the longer the shell-breaking time, and when the particle diameter reaches 100 μm, the shell-breaking time no longer increases.

## 4. Conclusions

In conclusion, the cause of shell-breaking was analyzed by simulating the pressure situation of Al particles, and the response time of shell-breaking for different particle sizes was obtained. And the thermo-mechanical behaviors of micron and nanometer Al particles were systematically summarized. The conclusions are as follows:

(1) The direct cause of shell-breaking of Al particles is clearly related to the particle size. The 25–100 nm Al particles showed no shell breakage, and all the particles of the diameter larger than 100 nm showed shell breakage. Among the Al particles of 200 nm–70 μm, except for the 300 nm Al particles where tensile stress overloading was the direct cause of shell breaking, the rest of them were directly affected by compressive stress overloading which broke the shell. And 80–100 μm Al particles break shells because of tensile stress overload. This result has never been reported in the literature.
(2) The thermal stabilization time increases with increasing particle size, and there is a significant difference in the shell-breaking response time between micron and nano Al particles. The shell-breaking response time of 200~600 nm Al particles is much smaller than that of 10~100 μm Al particles, which is 62.33~380.13 ps and 0.08~3.94 μs, respectively, which suggests that the Al nanoparticles are more susceptible to reaction under the same heating conditions.

## Figures and Tables

**Table 1:** Physical properties of Al and alumina.

Material	Melting Point (K)	Density (kg/m^3^)	Elastic Modulus (Gpa)	Bulk Modulus (Gpa)	Poisson’s Ratio	Thermal Conductivity (W/m·K)	Thermal Expansion Coefficient (K^−1^)
Al [27]	933	2700	70–79	76	0.35	210	23.0 × 10^−6^
alumina [28]	3729	3000–4000	300	165	0.21	18	8.6 × 10^−6^

**Table 2:** Comprehensive analysis of the shell-breaking response of Al nanoparticles of different sizes.

Particle Size (nm)	Shell Thickness (nm)	Ratio (R)	CS Time (ps)	TS Time (ps)	The Direct Cause of Structural Response
25	2.995	8.347	Un	Un	Un
30	3.043	9.859	Un	Un	Un
40	3.011	13.285	Un	Un	Un
50	3.007	16.628	Un	Un	Un
60	2.972	20.188	Un	Un	Un
70	2.991	23.404	Un	Un	Un
100	3.494	28.620	Un	Un	Un
200	3.838	52.110	62.33	78.97	compressive stress overload
300	4.044	74.184	107.52	94.36	tensile stress overload
400	4.092	97.752	122.78	190.58	compressive stress overload
500	4.227	118.287	198.29	240.69	compressive stress overload
600	4.275	140.351	380.13	400.93	compressive stress overload

Note: The time required for the pressure to reach the compressive strength is the compression shell-breaking response time, and the time required for the tensile stress to reach the tensile strength is the tensile shell-breaking response time. The ratio R is the ratio of the Al particle size to the thickness of the Alumina shell, and Un represents undamaged. All shell-breaking phenomena in Table 2 occurred on the outer surface of the alumina shell.

## Data Availability

The original contributions presented in the study are included in the article, further inquiries can be directed to the corresponding authors.
